# Smart Home Sensing and Monitoring in Households With Dementia: User-Centered Design Approach

**DOI:** 10.2196/27047

**Published:** 2021-08-11

**Authors:** Federico Tiersen, Philippa Batey, Matthew J C Harrison, Lenny Naar, Alina-Irina Serban, Sarah J C Daniels, Rafael A Calvo

**Affiliations:** 1 Dyson School of Design Engineering Imperial College London London United Kingdom; 2 Care Research and Technology Centre UK Dementia Research Institute Imperial College London London United Kingdom; 3 Institute of Global Health Innovation Imperial College London London United Kingdom; 4 Department of Brain Sciences Imperial College London London United Kingdom

**Keywords:** assistive technology, independent living, internet of things, remote monitoring, dementia, human centered design, user-centered design, patient-centered care, smart home, digital health

## Abstract

**Background:**

As life expectancy grows, so do the challenges of caring for an aging population. Older adults, including people with dementia, want to live independently and feel in control of their lives for as long as possible. Assistive technologies powered by artificial intelligence and internet of things devices are being proposed to provide living environments that support the users’ safety, psychological, and medical needs through remote monitoring and interventions.

**Objective:**

This study investigates the functional, psychosocial, and environmental needs of people living with dementia, their caregivers, clinicians, and health and social care service providers toward the design and implementation of smart home systems.

**Methods:**

We used an iterative user-centered design approach comprising 9 substudies. First, semistructured interviews (9 people with dementia, 9 caregivers, and 10 academic and clinical staff) and workshops (35 pairs of people with dementia and caregivers, and 12 health and social care clinicians) were conducted to define the needs of people with dementia, home caregivers, and professional stakeholders in both daily activities and technology-specific interactions. Then, the spectrum of needs identified was represented via patient–caregiver personas and discussed with stakeholders in a workshop (14 occupational therapists; 4 National Health Service pathway directors; and 6 researchers in occupational therapy, neuropsychiatry, and engineering) and 2 focus groups with managers of health care services (n=8), eliciting opportunities for innovative care technologies and public health strategies. Finally, these design opportunities were discussed in semistructured interviews with participants of a smart home trial involving environmental sensors, physiological measurement devices, smartwatches, and tablet-based chatbots and cognitive assessment puzzles (10 caregivers and 2 people with dementia). A thematic analysis revealed factors that motivate household members to use these technologies.

**Results:**

Outcomes of these activities include a qualitative and quantitative analysis of patient, caregiver, and clinician needs and the identification of challenges and opportunities for the design and implementation of remote monitoring systems in public health pathways.

**Conclusions:**

Participatory design methods supported the triangulation of stakeholder perspectives to aid the development of more patient-centered interventions and their translation to clinical practice and public health strategy. We discuss the implications and limitations of our findings, the value and the applicability of our methodology, and directions for future research.

## Introduction

### Background

Dementia is a syndrome that accounts for the ongoing decline of brain functioning including problems such as memory loss, thinking speed, mental sharpness, language, understanding, judgment, mood, movement, and difficulty in carrying out daily activities [1]. Around 50 million people have dementia, with 10 million new cases reported each year [1]. The psychological and physical impacts on patients, caregivers, and families can be devastating and life-limiting. The economic cost is also significant, costing over £30,000 (US $41,628) annually per person with dementia in the UK [2]. Because of the wide-ranging consequences of the illness, interventions need to address health, safety, and psychological concerns.

Understanding the needs of people living with dementia is critical and finding ways to support patient and caregiver autonomy and well-being is an ethical imperative. During the early stages of the illness most people with dementia want to remain living in their own home as independently as possible. In the advanced stages of the disease, psychiatric and behavioral disturbances are common, and patients often require professional medical care. Patients may suffer significant personality changes, hallucinations, paranoid ideas, aggression, wandering, and incontinence, so care is often provided in special facilities. In this study we focus on care in the home environment.

It is generally agreed that participatory approaches to research and development are essential for the design of products and services that satisfy patients’ health and psychological needs. Designers agree that user-centeredness helps create products that are more useful and engaging. Interventions should be designed based on a holistic understanding of the patient’s values, goals, functional abilities as well contextual factors such as living situation, relationships, and daily habits [3]. Including all stakeholders, not only people living with dementia and caregivers, can enrich participatory design activities.

This user research is particularly important when developing artificial intelligence (AI) and the internet of things (IoT) systems. On the one hand, they offer unprecedented opportunities to build environments that are safe for patients and caregivers and support autonomy and well-being. At the UK Dementia Research Institute (UK DRI), Care Research and Technology Centre (CR&T), advances in these technologies enable our smart home system to interpret the broad range of data input from devices and sensors in the home to infer behavioral, physiological, and cognitive markers and create alerts for human intervention (clinical or casual) through a cloud-based program [4-6].

On the other, AI, sensing, and monitoring also pose major potential threats. If they are taken as an unmitigated good and not carefully designed, they can have a significant negative impact on a disadvantaged community. This can go beyond basic requirements such as safety and accessibility. They can, for example, reduce the privacy and individual autonomy of patients and their families; they can be demeaning, unfair, or biased. Ethical and social risks are a significant barrier to a more widespread adoption of intelligent assistive technologies (ATs) for dementia. While concerns about autonomy are the most prevalent in literature, issues surrounding beneficence, justice, interdependence, and privacy have been identified [7]. A systematic review of the ethics of ambient assistive living technologies for people with dementia identified the involvement of patients in product development, informed consent, social isolation, and cybersecurity as sources of ethical risks [8].

More specifically, there is evidence that smart home technologies can be included in a pattern of elderly abuse (see, eg, [9]). This risk becomes particularly relevant for people with dementia as they may inherently be in a position where smart homes are used *on* them rather than *by* them. Moreover, their cognitive impairments may make them unable to provide informed consent to alterations of their privacy and agency [10]. Older adults have identified privacy issues surrounding smart homes [11], and the psychosocial impact of feeling under surveillance has privacy-related implications [12] that cannot be overlooked.

These factors must be investigated through the perspectives of end users (people with dementia and elderly caregivers) who may have very different expectations of these technologies due to their cognitive impairments or their cultural beliefs to those of the designers, engineers, and health care providers developing and implementing these technologies (eg, [13]). Such a multitude of perspectives can only be captured through participatory activities with the people directly involved (people with dementia and caregivers) and with clinicians who are experts at understanding the medical, psychosocial, and contextual needs of the people they care for. Moreover, older adults who have never tried smart home systems may have very different understandings of these technologies than people who have used them. People who have lived in smart homes express fewer concerns regarding intrusion, privacy, trust, and usability and more concerns about their utility [14]. Participatory activities should therefore investigate both actual use and anticipated use by involving current smart home users as well as members of the wider community.

This study aims to explore the development and translation of such opportunities while preventing such risks through participatory and user-centered design methods. We iteratively define and evaluate opportunities and challenges with end users (people with dementia and caregivers) and a wide range of stakeholders.

**Figure 1:**
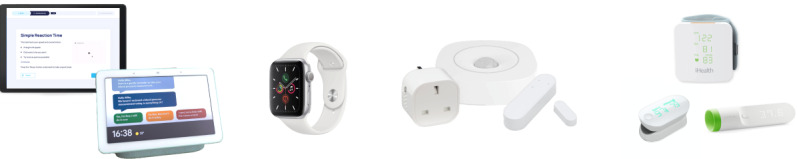
The internet of things technologies implemented in this study.

This study explores opportunities for care research and innovation enabled by the remote monitoring of data captured by the sensors illustrated in Figure 1. These include tablet-based cognitive assessment puzzles and chat interfaces, smartwatches, passive environmental sensors (appliances, bed, hallways, doors), and physiological measurement devices (blood pressure, blood oxygen saturation, temperature). These devices are used in households that are part of larger research program at the UK DRI CR&T. This paper describes design research studies conducted with the UK DRI CR&T cohort investigating their experiences of these technologies as well as studies with stakeholders from the wider public investigating needs in daily activities and health and social care.

### The UK DRI CR&T at Imperial College London and the University of Surrey, London

The CR&T aims “To empower people with dementia and their caregivers by using friendly ‘Healthy Homes’ - intelligent environments that transform and personalize care” [15]. To achieve this the CR&T is developing novel devices (including biosensors, point-of-care diagnostics, AI interfaces, sleep monitoring) which are monitored by a team of researchers and clinicians. The design and development team is highly interdisciplinary including dementia researchers, scientists, clinicians, interaction designers, and electronic, software, and design engineers at Imperial College London.

In this study researchers collaborated with end users and stakeholders including (1) trial participants of the wider UK DRI study [16] that had smart home systems installed in participants’ households collecting, analyzing, and intervening on behavioral and physiological data [4-6] (participants were originally recruited from local communities and associations to be representative of the dementia population and able to give informed consent); (2) patients, caregivers, and medics at the Imperial Memory Unit, Imperial College Healthcare National Health Service (NHS) Trust, Charing Cross Hospital; (3) the clinical monitoring team at the Surrey and Borders Partnership NHS Foundation Trust (SABP); (4) UK DRI CR&T members (clinicians and researchers); (5) clinical steering groups (clinicians and social workers); (6) people with dementia and caregivers who are members of the Alzheimer’s Society; (7) managers of the West London Frailty Services; and (8) service managers, neuropsychiatrists, clinical psychologists, and AT managers at the Hammersmith & Fulham Cognitive Impairment and Dementia Services, West London NHS Trust.

First, this study explores the needs of people living with dementia, home caregivers, and professional stakeholders (clinicians, researchers, and health care service providers) in both daily activities and technology-specific interactions. Based on those needs, we identify opportunities for care innovation in the broad design space enabled by emerging remote monitoring technologies. The final substudy then explores how to effectively translate some of these opportunities to clinical practice through user-centered design. We review existing literature in participatory design research for dementia care and provisions for research in this section. The design research methods used in this study are described in the “Methods” section. The “Results” section outlines the study findings, and the “Discussion” section discusses implications and concludes.

### Literature Review

#### Challenges in Designing Support Systems for Individuals With Dementia

Designing to support people with dementia is very challenging. First, there are declining cognitive and physical abilities that need to be addressed to reduce risks of illness and accidents. For example, preventable causes such as disease of the urinary system, pneumonia, and lower respiratory infections account for 20% of admissions to hospitals for patients with dementia, and another 16% is accounted by injury and poisoning [17]. But designers also need to consider other stakeholders such as the patients’ caregivers. This includes paid staff (eg, occupational therapists [OTs] and clinical teams) who are in short supply, and family members who are generally untrained, often find it difficult to deal with the strain of caring, and are at high risk of mental illness [18].

#### Designing for People With Dementia

Concerns about designing for people with dementia have been addressed in different ways. Some attempt to address the behavioral needs of people with dementia as defined by the literature as comprehensively as possible. For example, early studies on designing environments for people with dementia recommended that dementia-specific residential facilities should compensate for disability, maximize autonomy, and support personal identity, enhancing self-esteem [19]. A more recent study [20] takes a top–down system design approach and identifies stakeholders and use scenarios (eg, risk of dehydration, isolation, night-time wandering) from dementia care literature before defining opportunities for smart home touchpoints (eg, *inviting awareness* to drink, *performing* communication with acquaintances, *urging caregivers* to react to wandering episodes). The authors then involve caregivers and clinicians to qualitatively evaluate their use cases and to refine the system’s requirements.

Alternatively, involving people with dementia and caregivers in the design process can reveal more nuanced experiential factors. Orpwood and colleagues [21] discussed potential smart home features with caregivers and concluded that such systems should have familiar appearances and affordances, could incorporate verbal prompts and reminders, and should emulate caregivers when intervening to respect the person with dementia’s autonomy. For example, automated interventions should encourage the person with dementia to resolve the issue they forgot about (eg, “remember, you left the tap open”) before doing things for them to support autonomy rather than conveying helplessness [22]. However, notifying people with dementia, caregivers, or clinicians about every opening or closing of doors, taps, and appliances (eg, [23]) can be overwhelming. Machine learning can increase the precision of activity detection and help prioritize urgent medical and functional alerts [4].

Besides environmental sensors, passive sensing through ubiquitous devices such as smartphones and wearables can provide objective, rich, and granular data on clinically robust measures [24]. For example, a variety of daily activities (eg, boarding transport vehicles, washing dishes, or talking) of a person with dementia can be inferred from data sensed by his/her smartphones’ microphone and accelerometer [25]. Ubiquitous devices can achieve a context-bounded understanding of human activity, capture users’ attention when an intervention is needed, and otherwise “calmly” remain in the periphery of their attention [26]. It follows that, to maximize their benefits, such care systems should be designed focusing on the contextual experiences of patients rather than on the condition. The person with dementia should be considered “an active participant in everyday life rather than a passive recipient of care” [27].

#### Participatory Design Approaches

More recently, the call for attending to experience and researching and designing *with* rather than *for* users and stakeholders of health and social care services [28,29] received further attention in the human–computer interaction community. For example, Morrissey et al [30] explored the potential of collaborative, explorative, experience-centered design to more finely understand long-stay residential care experiences and design products that are more useful in that context. This new approach has prompted workshops with researchers, industry stakeholders, and communities of people with dementia [31] to further develop co-design processes for dementia-friendly ATs. They highlight the need for higher patient and clinician involvement in design research as both participants and leaders. Patients and stakeholders should also be involved in translating findings to industry to commercialize less “one-size-fits-all,” more personalized technologies, and to consider the impact and consequences of AT on how people with dementia engage with their communities [31].

Dementia-specific participatory design approaches are increasingly common. Topics covered include the design of long-term care environments (reviewed by Fleming and Purandare [32]) as well as interventions with context-specific purposes. For example, Houben and colleagues [33] explored therapeutic sounds with people with dementia and Jayatilaka and colleagues [34] investigated the challenges around people with dementia’s eating behaviors with care workers. Conducting co-design activities with a more comprehensive set of stakeholder groups can help design more user-centered care *services* in addition to single touchpoints or products. For example, Goeman and colleagues [35] involved people with dementia, care partners, aged-care service experts, policymakers, and academics to define the role for a new “key worker” in community settings. Moreover, investigations can be conducted in multiple stages to use optimal methods for each phase of iterative design processes. For example, while co-designing a novel IoT assistive product, focus groups may be used for scoping, workshops for product ideation, and interviews for prototype development and evaluation [36]. This approach led Robinson and colleagues [36] to identify tracking devices as stigmatizing, intrusive, and coercive before designing a smart armband that guides people with dementia home during wandering episodes without sharing their location with anyone else.

*Personas* can be co-developed with people with dementia to enable them to synthesize their needs and empathize with other potential users without directly confronting their personal relationship with their condition [37]. While developing a self-management smart home system for people with dementia and Parkinson disease, Bourazeri and Stumpf [37] used persona-based workshops to (1) explore the background, technology use, activities, and goals of users; (2) explore the use of sensors and gain input to the computational model; (3) design the user interface using low-fidelity prototyping; and (4) evaluate the interface design via cognitive walkthroughs. For example, a floorplan of a hypothetical home was used to allow workshop participants to envision possible uses of smart home sensors without being constrained by their personal living situations [37]. Furthermore, the input of other stakeholders from health and social care can complement patient co-design activities to ensure personas are representative of the spectrum of demographics, disease symptoms, needs, behaviors, and attitudes of their service users.

Achieving confidence and compliance with technological platforms that may be unfamiliar to an elderly population (eg, smartphones, tablets, IoT devices) requires designers to ensure accessibility, perceived privacy, and trust in both adoption and use [38]. For example, older adults may be especially wary about sharing personal information or obeying automated instructions, or they may perceive such devices as stigmatizing. Collaborative investigation therefore needs to reveal personal and social emotional aspects (eg, perceived confidence, dignity, independence) in addition to physical and cognitive impairments. Focusing on smart homes for elderly adults without dementia, Curumsing et al [39] advocated the need to include human, social, and organizational factors into smart home engineering. They systematically related the emotions experienced during use of a system (eg, anger, disgust, joy) to users’ underlying emotional *expectations* when adopting the system (eg, the elderly feeling cared for and independent, and caregivers feeling reassured). Capturing, representing, and evaluating both functional and emotional goals of elderly adults, caregivers, and relatives across all touchpoints and use cases resulted in a smart home system that alleviates health concerns and loneliness and is perceived by end users as empowering, caring, safe, and neither controlling, stigmatizing, nor intrusive.

Collaborating with older adults can be particularly beneficial for designers, as they “often challenge simplistic technological solutions to complex problems and help us question and critique the values and ethics embedded in the technologies we set out to design” [40]. For example, Ghorayeb and colleagues [14] qualitatively evaluated smart home systems both with older adults living independently in their communities and with participants who had been living in smart homes for 8-12 months. Anticipating the use of a technology that may be of future rather than current value to them led the first group to express concerns about the technology being intrusive, noticeable, and increasing the household’s vulnerability. Reflecting on actual use led the second group to be more critical about smart homes’ utility but less weary about privacy, trust, and usability. Both groups suggested making functionality customizable and shared concerns about smart homes’ affordability, their impact on relationships, and about the engagement and competencies of those monitoring their data. To capture this variety in perspectives, this study’s sampling strategy should include both members of the public and people who have *decided* to have a smart home installed and have experience living in it.

#### Designing for Patient-Centered Care

Designing *with* rather than *for* patients with dementia maximizes the benefits of specific technologies [28] as well as of programs of clinical care [41]. A shift in philosophy from traditional medical models of care that focus on processes, schedules, and staff and organizational needs to *person-centered care* was pioneered by Kitwood [42]. He conceptualized dementia as the interplay between neurological impairment and psychosocial factors including the individual’s health, psychology, environment, and social context.

Operationalizing person-centered care requires establishing interpersonal relationships with people with dementia and caregivers to identify and address the needs of individuals, as well as commitment from everyone within care organizations, especially leadership [41]. Similarly, creating technologies that support person-centered health care requires designers to personally empathize with patients to understand the experience of living with specific conditions and the concerns and emotions of vulnerable participants [43].

However, when designing for a variety of stakeholders and analyzing data in which one group speaks for another group, care must be taken to verify whether the second group actually disagrees. This phenomenon has been discussed by Cajander and Grünloh [44] and can be mitigated by a careful triangulation of data sources [45]. We achieved this through *value-sensitive design*, a theoretical and experimental framework comprising techniques to investigate stakeholders’ values and relationships around a common phenomenon to uncover innovation opportunities and manage value tensions through design [46]. In this study, the phenomena being investigated include interactions with remote monitoring technologies as well as, more broadly, life and care with dementia.

Designing *with* rather than *for* users becomes particularly important when creating products and services for people with dementia [28] because they inherently have very different experiences and abilities from those of the designers, engineers, clinicians, and researchers who develop such clinical tools [47]. Capturing these differences in mental models, however, comes with significant ethical and logistical challenges. The work by Waycott and Vines [40] on research ethics with older adults addresses issues around beneficence, justice, respect, and research merit and integrity [48].

The integrity of the research could be compromised, for example, if an episode of cognitive decline leads a participant with dementia to misinterpret the researcher as a loved one and thus affects their ability to provide informed consent and alters power balances. Ethnographic activities and interviews involving people with dementia in this study therefore always involved the accompaniment of their principal caregiver.

In addition to providing insight into their personal needs as stakeholders of smart home systems, working with caregivers and clinicians with expertise in the needs of people with dementia as “surrogates” for patients can enable researchers to bypass some of these logistical and ethical challenges and to achieve an understanding of people with dementia’s needs more efficiently. The involvement of stakeholders in this study should nevertheless *complement*, not *replace*, that of people with dementia. Bartels and colleagues [49] found that people with mild dementia retain the ability and insight to accurately reflect on their own ability to use everyday technologies. Complementing self-reports on the use of technologies in an individual’s everyday life with the observation of specific interactions with technology and the consideration of underlying psychological determinants thus leads to a more thorough understanding of patients as individual technology users. The perspectives of other stakeholders can therefore add value in interpreting self-reported and observed needs to build a more thorough understanding of the complex, dynamic, and comorbid needs of people with dementia. This becomes especially valuable when the disease’s progression may impair the cognitive abilities required to perceive, recognize, and express such needs.

Envisioning intangible concepts, maintaining structure in meetings, and preventing stigmatization are common challenges in designing with older adults [50] or vulnerable people [51]. Prolonged discussions about abstract concepts are particularly challenging to people with dementia due to their cognitive impairments [52] and possibly distressing due to the confrontation with their disabilities [53]. Self-expression should be encouraged by focusing on the abilities of the person with dementia (eg, interacting with tangible objects, creating, sharing) rather than on their deficits [54]. Cocreation activities that are aligned to all participants’ abilities and that allow them to express their individuality can be beneficial to people with dementia as well as designers. Successful activities can help recently diagnosed patients to build their self-esteem, identity, and dignity and can help keep them connected to their community [55].

## Methods

### Overview

Functional and psychological human needs, and social and organizational factors, should be addressed through human-centered design approaches that create empathy with users (people with dementia and their principal caregivers) and stakeholders (clinicians, researchers, and health care service managers). Our approach to user research, and building such empathy, is through home visits, shadowing and observation, workshops, and in-depth interviews with a diverse range of representative users and stakeholders. Participants included people with dementia, home caregivers, clinicians (OTs, clinical psychologists, nurses), social workers, managers of cognitive impairment and frailty-related public health care services, and researchers in health and technology.

Such research activities informed the creation of personas that represent the spectrum of needs and aspirations of the intended users. A thematic analysis revealed factors affecting acceptance of and engagement with AT as well as challenges and opportunities related to their implementation.

We used a mixed methods approach including semistructured interviews, focus groups, workshops, and ethnographic observation (shadowing). The latter informed the process but is not reported due to incomplete documentation. Each of the methods was applied to end users (caregivers and people with dementia) and stakeholders (clinical, research, and health care service management teams). Interviews, visits, and workshops were carried out by researchers at the Helix Centre, the UK DRI CR&T, and the Dyson School of Design Engineering and approved by the Human Research Ethics Committee of Imperial College London.

**Figure 2:**
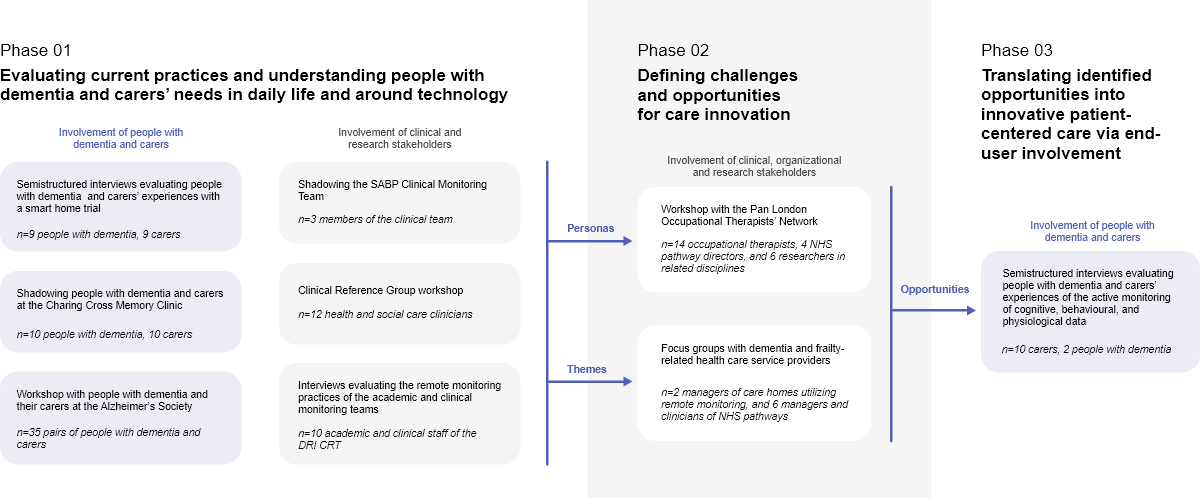
Purpose and activities of each of the three phases of this study. CR&T: Care Research and Technology Centre; DRI: Dementia Research Institute; NHS: National Health Service; SABP: Surrey and Borders Partnership.

Having different researchers (1 to 3 of the authors ran each substudy) conducting a variety of methods to gain input from various users and stakeholders resulted in the triangulation of investigators, methods, and data sources [45] to develop a more comprehensive understanding of the phenomena being studied. The diversity of methods and stakeholders involved in this study allowed researchers to alternate divergent and convergent investigations. The tripartite approach illustrated in Figure 2 enabled researchers to iteratively develop a thorough understanding of the design space surrounding dementia life and care and, more specifically, interventions enabled by remote-monitoring technologies.

Generally, the first phase of this study focused on evaluating people with dementia and caregivers’ experiences of daily activities, clinical visits, and a smart home system. Additionally, this phase investigated clinicians’ experiences of supporting such daily activities through such clinical and social care appointments and pathways as well as smart homes. The generalization of these findings informed the definition of a rich set of personas that not only include the person with dementia but also his/her principal caregiver.

In a more generative second phase, these personas were used as case studies to elicit a more comprehensive set of needs, frustrations, and opportunities from the perspective of OTs, health care managers, and researchers. Focus groups with dementia and frailty-related health care service providers explored related topics from the perspective of a wider range of stakeholders. This phase resulted in the definition of a set of challenges and opportunities for innovation.

Finally, more focused interviews with users (people with dementia and home caregivers) around their experiences of a more intensive remote monitoring system enabled a deeper validation and exploration of some of the challenges and opportunities defined in the second phase from the perspectives of people with dementia and caregivers. Namely, this smart home system involved (1) implementing remote cognitive assessments; (2) educating patients and caregivers to use proposed technologies; (3) identifying and addressing causes of psychological disturbances related to interventions; (4) collecting objective behavioral and physiological data; and (5) providing reliable clinical oversight to manage false alarms and prevent anxiety. This third user-centered design phase enables opportunities that were defined by clinicians in *Phase 02* based on *Phase 01*’s findings to be developed into accessible, usable, useful, and desirable products that can be successfully translated in clinical practice.

### Semistructured Interviews

Three sets of semistructured interviews were performed.

#### Evaluating People With Dementia and Caregivers’ Experiences With a Smart Home Trial

First, we visited 9 homes of participants who had experienced the smart home technologies as part of the UK DRI trial. The interviews occurred during visits (1-2 hours long) and included semistructured conversations around themes within the larger project with 9 people with dementia and 9 caregivers. Discussions include guided observations of people with dementia and their caregivers within their home environment to help the design process by getting feedback on future design solutions.

**Figure 3:**
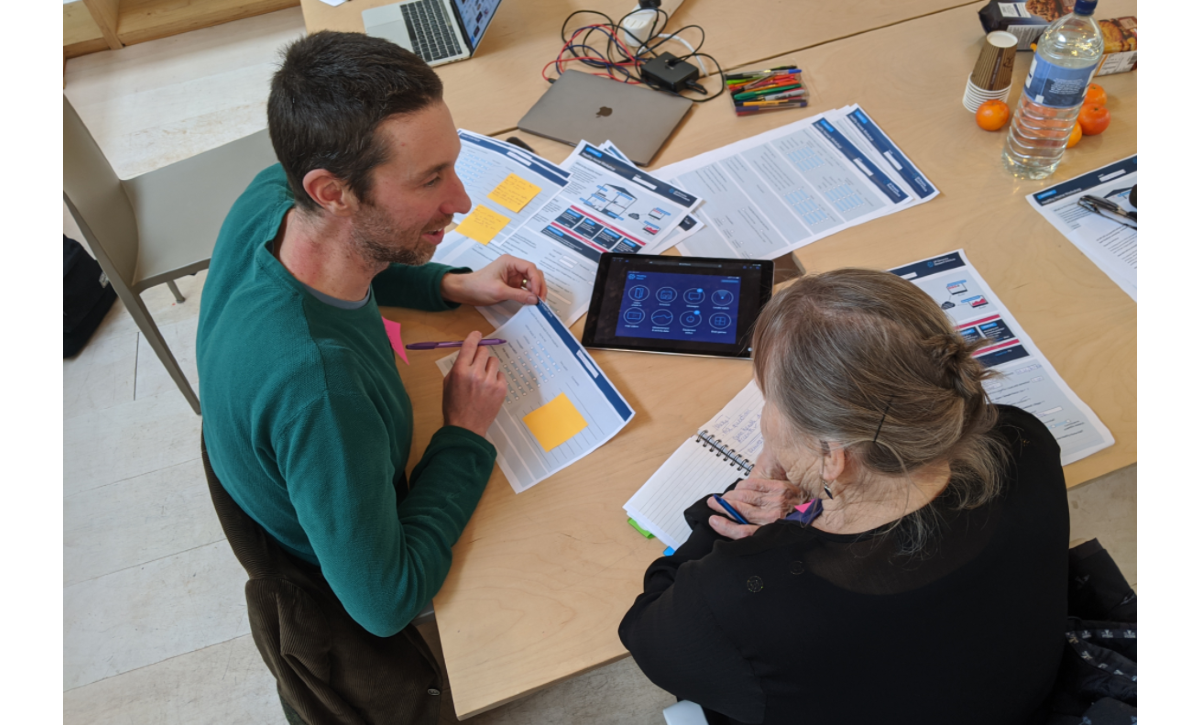
Exploring the needs surrounding proposed smart home touchpoints with a home caregiver.

Participants were then invited to the Helix Centre to evaluate proposed features that were being considered for the center’s smart home system. The Helix team used rapid cycles of “provocative prototyping” with multiple low-fidelity concepts of smart home interactions. This elicited end user needs specific to particular technologies and allowed to steer the focus of technological development at regular intervals to promote creative problem solving. Figure 3 illustrates this activity.

#### Evaluating the Current Remote Monitoring Practices of the Academic and Clinical Monitoring Teams

In the second set of semistructured interviews, 2 design researchers interviewed 10 academic and clinical staff from the UK DRI CR&T. Interviews were intended to provide a different perspective to that of users. Their academic training, their expertise with methods aimed at improving people’s health, and their experience caring for others could frequently allow them to find patterns of problems and solutions. People with dementia and caregivers had highlighted that an important factor of patient engagement is the connection they make with this team.

#### Evaluating Persons Living With Dementia and Caregivers’ Experiences of the Active Monitoring of Cognitive, Behavioral, and Physiological Data

The third set of semistructured interviews (10 caregivers, 2 people with dementia; 20-50 minutes per interview) was conducted in 10 households with patients and caregivers who trialed a remote monitoring device and cognitive test battery comprising a smartwatch, a tablet, a pulse oximeter, and a thermometer for 2 weeks. Restrictions imposed by the COVID-19 situation led researchers to conduct these interviews via phone calls. Contrary to home visits observing and discussing in situ interactions with technologies, this medium relies on memory, self-reporting, and abstraction, and thus excluded 8 moderate and advanced patients with dementia from being active participants in these interviews. This substudy explored some of the opportunities elicited in Phase 02. A thematic analysis revealed factors that can motivate or disengage users when adding more active or intrusive products into a passive smart home configuration.

### Focus Groups With Health Care Service Providers

Two group discussions were held through online videoconferencing software with stakeholders of 2 health care services. First, 2 managers of the West London Frailty Services discussed their experiences with remote physiological and activity monitoring in care homes. Discussions covered relevant topics including patient compliance with wearables, assigning responsibility for out-of-hours clinical monitoring, and information sharing between support services. Second, 6 stakeholders from the Hammersmith & Fulham Cognitive Impairment and Dementia Services, West London NHS Trust discussed opportunities and challenges in designing remote cognitive assessment products and ways to collaborate to design more inclusive services.

### Workshops

Three workshops were carried out with different groups to understand the needs of stakeholders within the smart home trial and the wider dementia context.

#### Clinical Reference Group Workshop

A group was set up to ensure the researchers gain insight from a range of clinicians within health and social care (n=12). Throughout the workshop, the group collaboratively generated a map of 19 needs that are common to people with dementia from different perspectives, then went on to plot 3 contrasting dementia journeys (from diagnosis to end of life care) to show how a person with dementia and his/her principal caregiver would navigate through the UK’s health and social care system.

#### Workshop With People With Dementia and Their Caregivers at the Alzheimer’s Society

**Figure 4:**
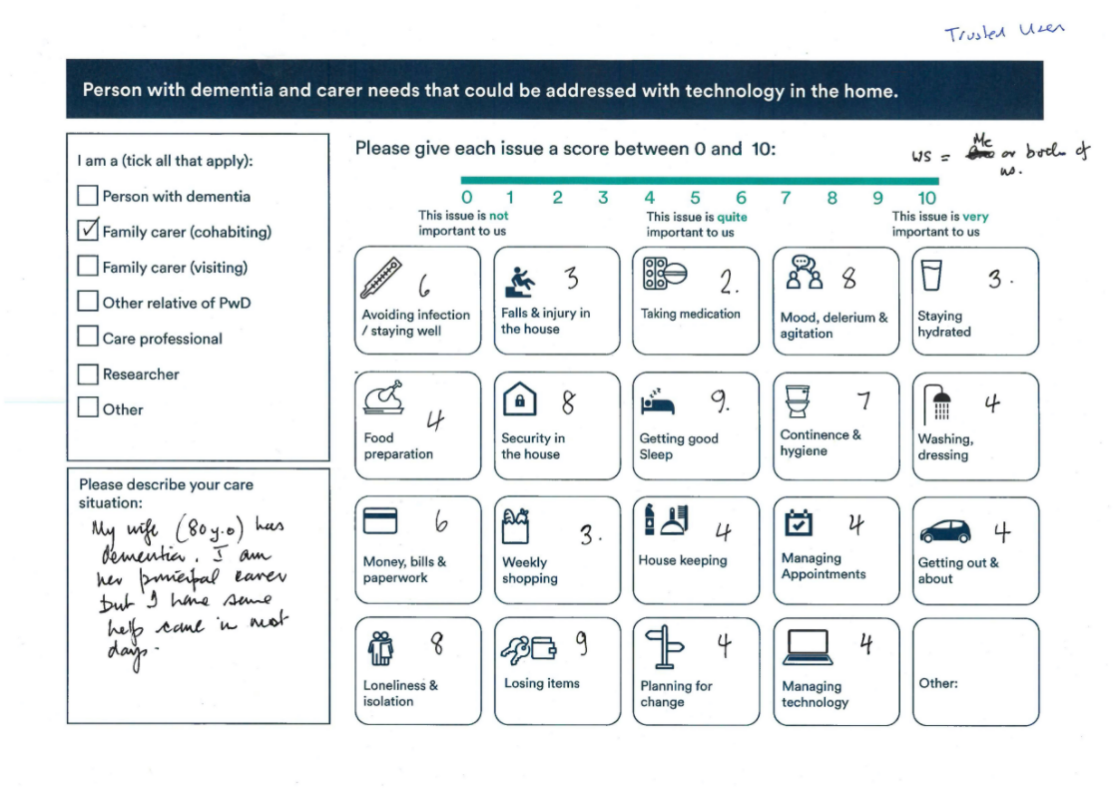
Needs map ranking worksheet.

A sample of people with dementia and caregivers that does not comprise early adopters of the CR&T’s remote monitoring systems and is therefore more representative of the general population was selected to investigate the prevalence of needs in activities of daily living (ADLs) in dementia households. As part of a workshop at the Alzheimer’s Society in London, pairs of people with dementia and caregivers were asked to complete a worksheet scoring their needs (Figure 4) on parameters defined in the previous workshop with the *Clinical Reference Group*, and 35 responses were received. The worksheets identified and prioritized the perceived needs of individuals in various aspects of daily life affected by dementia to help ensure that the interventions of the smart home system would address the most pressing concerns of people with dementia and caregivers.

#### Workshop With the Pan London Occupational Therapists’ Network

OTs’ clinical roles and the similarities between user-centered design and occupational screening [3] make OTs suitable for participatory design activities aimed at (1) understanding the needs of clinical monitoring teams as service providers and users of remote monitoring technologies; (2) defining the spectrum of care needs of their patients and their caregivers; and (3) making the scenarios (personas) ideated by design researchers more clinically relevant and comprehensive.

A workshop with 24 participants (14 OT; 4 NHS pathway directors; 6 researchers in occupational therapy, neuropsychiatry, and engineering) was hosted online through Zoom, Miro, and Qualtrics at a conference held by the UK DRI CR&T for the *Pan London Occupational Therapists’ Network*. Because of the nature of their roles, multiple members of the same multidisciplinary teams cannot take half days off to participate in an in-depth workshop synchronously. Alternating between group calls and 8 breakout rooms in Zoom allowed for parallel discussions and contributions to maximize efficiency and limit the workshop to under an hour. Qualtrics was used to record asynchronous inputs around discussed topics both before and after the session. Subjects covered include challenges and frustrations when delivering their services, use cases of specific ATs, service changes imposed by COVID, factors affecting the deployment of assistive products, and wished-for technology developments.

The patient–caregiver personas described in this paper were used as case studies to systematically elicit specific desires and concerns while assessing, treating, evaluating, and discharging patients. Wearables, remote physiological and behavioral monitoring, and virtual communication technologies were explored as solutions. For each case study, participants were separated into groups of 3 in their breakout rooms to contribute their desires (eg, answers to “if technology could let you see or do anything about this person, what would you like to see or do? Why?”) and concerns (eg, answers to “do you foresee any problems or barriers to implementation? Why?”) to the aforementioned categories in the Miro board. Breakout rooms increased the number of contributions by enabling 8 parallel conversations where all attendees are prompted to actively participate. All participants regrouped at the end of each case study to share inputs and triangulate results. The “Patient–Caregiver Personas” section illustrates these case studies, while the “Current Challenges in Delivering Professional Care Identified by Clinicians, Researchers, and Health Care Managers” and “Technology and Service Development Opportunities Identified by Clinicians, Researchers, and Health Care Managers” sections illustrate this workshop’s outputs.

## Results

### Overview of Outcomes from Different Activities

The interviews, focus groups, and workshops produced useful insights about the users and their needs that we summarize here. The outputs of Phase 01 activities that preceded the definition of patient–caregiver personas were analyzed by transcribing key themes arising from interviews, observations, and workshops. Themes were organized into affinity diagrams in collaborative design workshops at the Helix Centre to identify patterns of end-user or stakeholder needs across all use cases. Together with *needs mapping*, these activities elicited a comprehensive understanding of personal experiences that helped define personas for use in further studies to design products and services that better address these needs. Moreover, the interactions that were observed between users and the monitoring team pointed to many of the design and usability issues within the current configuration of the UK DRI CR&T’s smart home system.

The second and third phases of this study build on findings of the first phase through their communication as personas and themes. Phases 02 and 03 were aimed at further exploring and defining challenges and opportunities in delivering technology-enabled care through the OT workshop, the 2 focus groups, and the last set of semi-structured interviews.

The audio from interviews and focus groups was recorded and fully transcribed using Descript (Descript, Inc.) and workshop outputs were exported from Miro and Qualtrics. A thematic analysis of all transcripts and workshop contributions was conducted by researchers using the coding and referencing software NVivo (QSR International)*.* An *inductive* analysis as described by Elo and Kyngäs [56] was conducted to derive concepts from the data. The analysis investigated everyday living and interactions with technology from a phenomenological perspective, focusing on participant’s subjective experiences of trialed or proposed technologies. The coding process involved 3 stages but was iterative in nature. First, researchers read the entire body of texts and defined a codebook of all the themes that emerged while coding the evidence with the newly defined themes in NVivo. Instances in which the theme being discussed could encapsulate other themes that had emerged prompted researchers to define layers of subthemes and reflect this architecture in NVivo. For example, the need to “establish duty of care” in public health services’ strategy contained “clinician stress,” “determining the appropriateness of episodic or continuous monitoring,” “understaffing,” and “handling urgent out-of-hours data” among its subthemes. Layers of meta-themes were also established to organize and communicate findings. The subthemes above were assigned to “lack of resources, infrastructures or information” under “current challenges in delivering professional care.” Findings from this thematic analysis were communicated both in prose for qualitative insights or in a table containing the number of instances in which a theme was mentioned toward a more quantitative understanding of the prevalence of different needs.

### Patient Needs as Mapped by Clinicians and Researchers and Prioritized by People With Dementia and Caregivers

Table 1 presents the breakdown of user responses from a mapping exercise held at an event for people with dementia and caregivers hosted by the Alzheimer’s Society. The categories of patient needs had been defined by the Clinical Reference Group workshop and their relative importance scored by people with dementia and their caregivers in the subsequent Alzheimer’s Society workshop. The sample included 35 people with dementia at various stages of disease progression and 35 principal caregivers. Each pair of people with dementia and their caregivers provided 1 set of responses via the needs mapping worksheet illustrated in Figure 4.

This analysis of patient needs suggests that preventing illness and injury is the most salient concern. Sleep, hydration, continence, hygiene, and psychological states are relevant targets for interventions. Medication compliance is also worthy of consideration.

This activity enabled researchers to start identifying and prioritizing areas of opportunity for intervention and to communicate a comprehensive spectrum of patient needs in the personas that were being defined. The clustering of needs (eg, correlations between infection and hydration, or between security and losing items) informed the definition of personas described below. Future needs mapping activities can analyze the impact of the patient’ stage of disease progression on prioritized needs.

**Table 1:** Needs of people with dementia as scored by 35 pairs of people with dementia and caregivers.

Needs map item	Cumulative score	Average^a^ (SD)	Completion
Avoiding infection, staying well	280	8.0 (3.0)	35
Falls and injury at home	269	7.9 (3.1)	34
Getting good sleep	269	7.9 (2.7)	34
Staying hydrated	269	7.7 (3.1)	35
Continence and hygiene	268	7.7 (3.1)	35
Mood, delirium, agitation	254	7.7 (3.2)	33
Taking medication	244	7.4 (3.6)	33
Washing and dressing	231	7.0 (3.0)	33
Loneliness and isolation	223	6.6 (3.6)	34
Losing items	218	6.2 (2.9)	35
Security in the house	207	6.3 (3.9)	33
Food preparation	185	5.8 (3.6)	32
Managing appointments	185	5.6 (3.6)	33
Getting out and about	177	5.7 (3.9)	31
Planning for change	175	5.6 (3.3)	31
Money, bills, paperwork	155	5.0 (4.1)	31
House keeping	136	4.0 (2.9)	34
Managing technology	117	3.9 (3.0)	30
Weekly shopping	114	3.8 (3.2)	30

^a^Blanks ignored.

### Patient–Caregiver Personas

#### Brief Overview of Personas

Personas (fictionalized representations of observed people) are a tool commonly used within human–computer interaction. Concepts and ideas can be tested against the expected requirements of each persona as an aid to ensuring the ideas are accessible to as many people as possible. The use of personas does not replace subsequent user testing, but they can be used in the early stages of product development as part of the creative process, and to communicate the breadth of user requirements to other collaborators within the technology development teams or in participatory design activities with service providers such as this study’s OT workshop.

The personas defined below comprise the spectrum of daily activity needs outlined in the previous section as well as psychosocial and contextual factors identified in *Phase 01* of this study. Researchers analyzed patterns and clusters in qualitative findings and generated affinity diagrams to define the personas. Furthermore, our engagement with a range of different stakeholders supported not only the prima facie content of a persona but also what elements are included within the persona. In the context of this project, we found that describing personas as a combined unit of patient and caregiver was more valuable in representing a meaningful situation. We also described a situation where there is no family caregiver as one of the personas. The description of each persona includes (1) engagement—how much the patients and caregivers interact with the technology, data, and the clinical monitoring team and why; (2) support needs—clinical and social care needs; (3) socioeconomic factors; (4) living situation; (5) support network; (6) habitual use of technology; (7) hobbies and daily activities; (8) main issues and challenges—the main health needs and the barriers to interacting with care providers and the smart home.

The authors identified 6 personas that combined traits of the people interviewed and their context but deliberately omitted the wide range of clinical and social care services that are delivered to patients. Focusing only on environments, patients and caregivers at this stage allowed researchers to use personas as open frameworks to guide workshops with the complex network of clinical and social care stakeholders. Pain points and desires were defined systematically and comprehensively to make technologies and interventions inclusive to all patients and use cases. Interviewees described requirements in ways that can be interpreted as needs for autonomy, competence, and relatedness. This is an area for further exploration. The 6 personas with fictitious names and homes identified are described and displayed below.

#### Alone Together (Betty and Husband)

**Figure 5:**
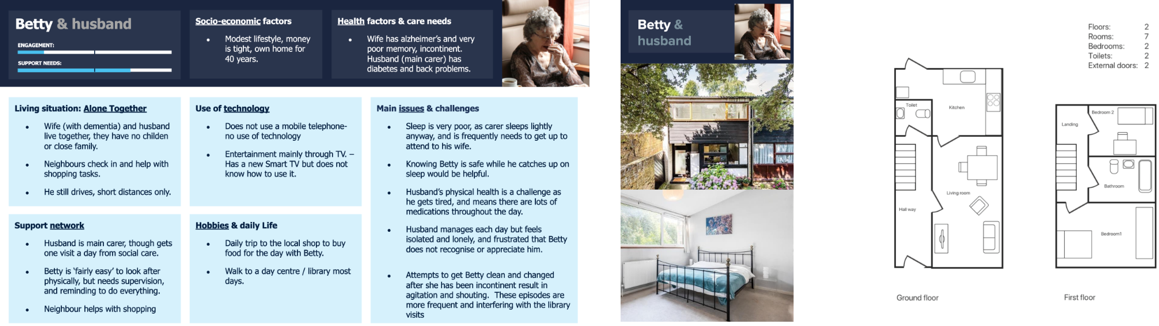
Persona A: Alone together.

Betty and her husband (Figure 5) live in a quiet house and have a large amount of time available to participate in the smart home trial. They both suffer from declining physical health which results in high care needs. Betty’s husband feels socially isolated which puts a strain on their relationship.

#### Supported Partnership (Aaron and Wife)

**Figure 6:**
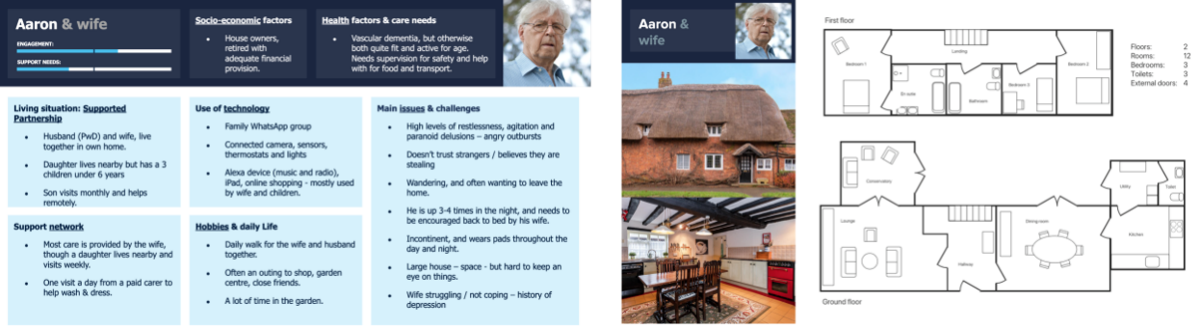
Persona B: Supported partnership.

Aaron (Figure 6) has high levels of support from his wife, neighbors, and community, lives in an affluent area, and has plenty of time available to engage with technology. Their big house raises challenges with device implementation. His technical skills mean he may be slower in learning to use devices and take measurements.

#### Evenings and Weekend (Carly and Daughter)

**Figure 7:**
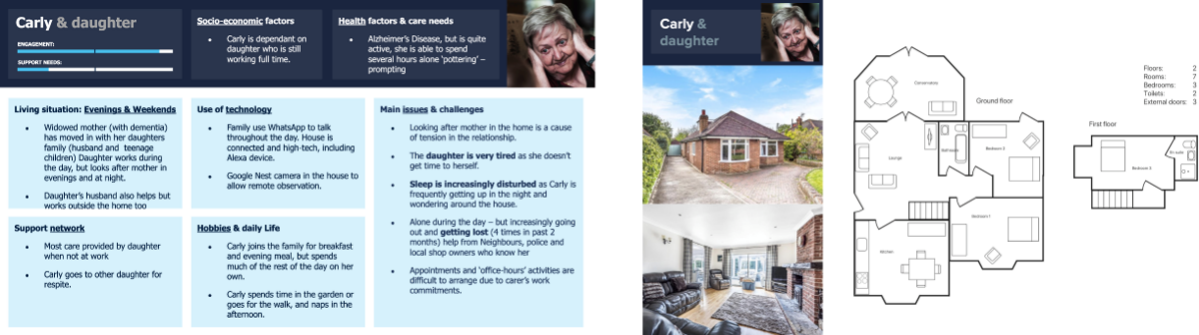
Persona C: Evenings & weekend.

Carly (Figure 7) has recently moved in with her daughter who looks after her in evenings and at night. Carly’s daughter and family are very tech savvy and can easily engage in the technology. Because of the nature of their living situation, Carly has restricted hours of support which causes her family to worry. Her families sleep is increasingly disturbed as Carly is frequently getting up in the night and wondering around the house.

#### Remote Relative (David and Son)

**Figure 8:**
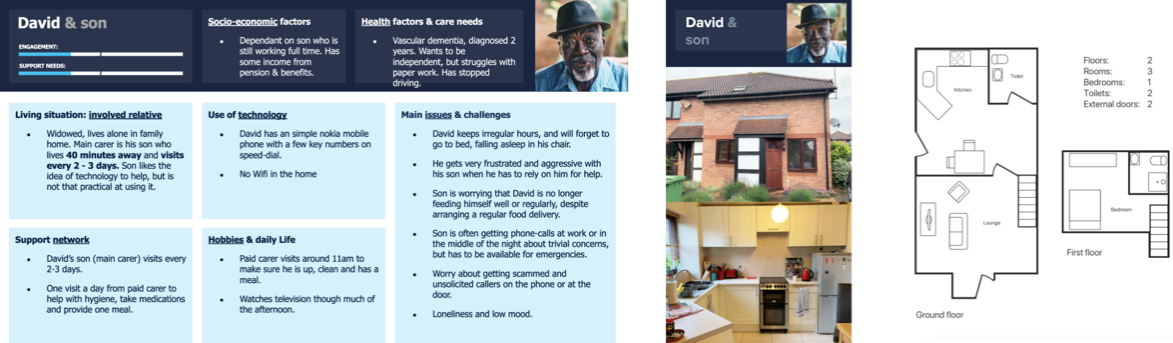
Persona D: Remote relative.

David (Figure 8) is a single father who lives alone. His son lives 40 minutes away and visits every 2-3 days. Being a single occupant in the house makes it easier for the technology to monitor behavior. David suffers from agitation and is reluctant to receive help from technology or other people. His son is only partially engaged.

#### Busy Home (Emily and Family)

**Figure 9:**
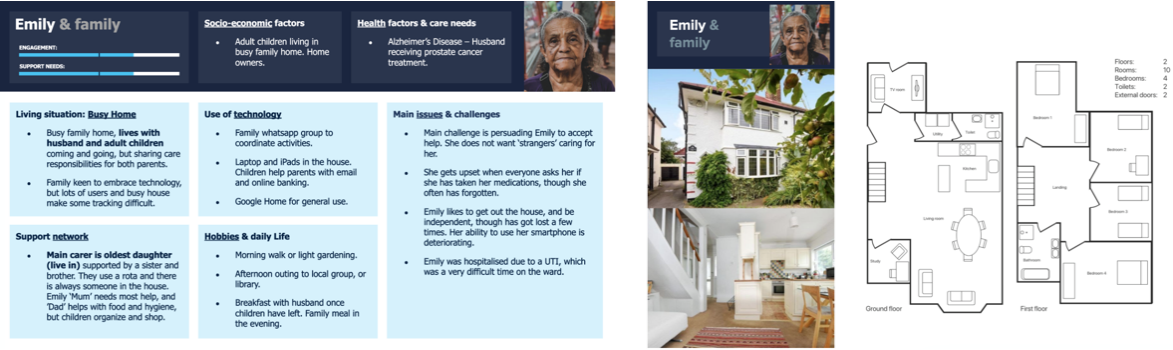
Persona E: Busy home.

Emily (Figure 9) lives in a busy home with her family who share the care responsibilities. The family is very keen to embrace technology and engage in the trail; however, lots of users and a busy house make monitoring behavior and managing care difficult.

#### Isolated Single (Fran)

**Figure 10:**
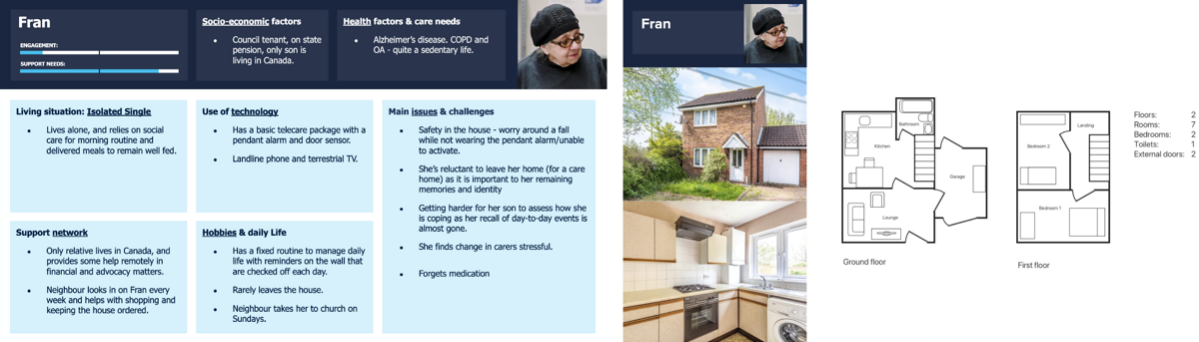
Persona F: Isolated single.

Fran (Figure 10) lives alone and relies on social care and delivered meals to remain well fed. She has many different paid caregivers for quick visits, which means she suffers from isolation. She has low technology engagement and worries about her safety in the house (eg, a fall that remains undetected).

Personas were later used in the *Pan London OT Network* workshop to communicate the needs of people with dementia and caregivers to health care stakeholders to prompt them to consider a more comprehensive set of situations while defining the problems faced in clinical privacy and the ways technology can support their care.

### Current Challenges in Delivering Professional Care Identified by Clinicians, Researchers, and Health Care Managers

#### Overview of Challenges

This section summarizes the pain points highlighted by clinicians, researchers in related fields, and managers of health care services in semistructured interviews, focus groups, and the *Pan-London OT Network* workshop. Although these challenges have been defined by stakeholders rather than end users (people with dementia and caregivers), these 3 substudies included the communication of end-user needs to said stakeholders via the *themes* and *personas* the authors defined in previous substudies. Moreover, involving this variety of stakeholders revealed factors that are representative of general public health scenarios and not limited to the CR&T’s early adopters of smart home systems.

#### Lack of Resources, Infrastructures, or Information

Access to ATs is not uniform across London services due to limited funding, availability, or misalignment with their patients’ needs. Information about latest innovations is not always readily available. It is common for IT systems to be unreliable and for data to not be accessible across support services. Limited staffing often forces teams to reduce focus on occupational performance to work on generic assessments and provide basic care. Continuous clinical monitoring is particularly challenging and raises ethical questions: round-the-clock monitoring is resource-intensive and can be detrimental to clinicians’ stress, while episodic monitoring may not be the best option for certain scenarios. There are ethical questions around duty of care and data being generated out of hours that could indicate an urgent clinical need. Some of our clinical participants opted to turn monitoring devices off at night.

#### Usability, Acceptance, and Consent

The lack of internet connection in patient homes and of funding for caregivers and family member to purchase assistive or communication devices are frequently coupled with skepticism or low abilities to engage with digital products. Similarly, it is common for patients to be reluctant to respond to automated alerts or notifications or to be monitored by sensors. If the perceived value of being monitored does not exceed the burden of participation, then alert fatigue and frustrations with devices may cause the participants to disengage. Many that could benefit from remote monitoring are isolated and lack mental capacity to understand its usefulness or to consent, and disengaged families may not agree with what clinicians suggest as the patient’s best interest.

#### COVID-19 Lockdown-Related Challenges

Building therapeutic rapport and completing functional assessment are more challenging without face-to-face contact, and increased isolation has led to the deconditioning and deterioration of many patients. By contrast, this context increases the importance and the rate of implementation of remote monitoring. Despite the heightened need, social distancing has also enhanced the challenges of providing technical support to install and maintain devices and of providing in-person training.

### Technology and Service Development Opportunities Identified by Clinicians, Researchers, and Health Care Managers

Opportunities for the design and integration of assisting technologies were identified and prioritized in Phase 02’s workshop and focus groups by OTs, neuroscience researchers, clinical psychologists, health care service leaders, and care home managers through open questions (eg, “what advances in technology would you like to see in the next five years?”, “what would you like to know or do [in this case study] if technology could let you know or do anything?”). Although no end users were involved in the definition of these opportunity areas, prompting stakeholders’ ideation with the themes and personas the authors defined in previous substudies has elicited great variety of ideas based on a more comprehensive consideration of end-users’ needs. Table 2 outlines the different categories and the number of instances in which they were mentioned.

**Table 2:** Technology and service development opportunities identified in a workshop with clinicians, researchers, and service managers and 2 focus groups with health care service providers.

Technology and service development opportunity	Mentions
Efficient, accurate remote cognitive assessments which are validated against standard tests despite learning, language, education, and cultural variations in patients	23
Objective covert behavioral and physiological data (eg, falls risk)	22
Measuring and managing caregiver strain through peer and professional support regarding dealing with situations, knowing what to expect, and planning for emergencies	16
Improving access to the wider network of casual and professional care and social services	15
Alternating between continuous and episodic measurements for optimal use of resources	8
Increased on-demand communication for practical, clinical, and emotional support	7
Informal monitoring products (eg, trackers) for caregivers	7
Educating patients and caregivers to use proposed technologies	6
Proactive medical interventions (eg, UTI prediction) to prevent further deterioration	6
Identifying and treating causes of psychological disturbances (eg, surveillance paranoia) before implementing intervention	5
Automated reminders and interventions supporting activities of daily living	5
Providing reliable clinical oversight to manage false alarms and prevent anxiety	5
Dynamic adjustment of medication administration enabled by granular monitoring of its effects	3

The most frequently mentioned desired technology development opportunities are related to unearthing novel, more accurate, objective data about cognitive, behavioral, and physiological parameters to enable clinicians to perform more informed assessments and distinguish between subtly different conditions (eg, between memory, language, visual-spatial, and sensory-motor deficiencies) in their diagnostics. Improving the availability and the quality of support and reassurance to caregivers through clinical, professional, and casual services and through informal care products and automated interventions is another priority. Having a platform over which to conduct intensive monitoring on an episodic basis can help treat acute conditions, counteract deterioration of preventable infections, and titrate drug prescriptions. Educating patients and caregivers about their prospective products and treating potential causes of rejection can improve compliance.

### Factors Affecting Compliance and Engagement With Active, Passive, and Intrusive Devices Identified by Exploring Phase 02’s Challenges and Opportunities With People With Dementia and Caregivers

#### Phase 03 Findings

Our Phase 03 interviews investigated 5 opportunities identified in the “Technology and Service Development Opportunities Identified by Clinicians, Researchers, and Health Care Managers” section from the perspectives of end users: (1) implementing remote cognitive assessments; (2) educating patients and caregivers to use proposed technologies; (3) identifying and addressing causes of psychological disturbances related to interventions; (4) collecting objective behavioral and physiological data; and (5) providing reliable clinical oversight to manage false alarms and prevent anxiety.

A thematic analysis of discussions with people with dementia and their home caregivers regarding the addition of pulse oximeters, thermometers, tablet-based cognitive testing puzzles, and smartwatches into the homes of selected study participants revealed factors that can motivate or disengage users. Achieving a deep understanding of such factors is crucial toward translating these technology-enabled opportunities into clinical practice.

#### Preventing Anxiety and Frustration

When dealing with sensitive data such as physiological readings and cognitive assessment results, any misunderstanding or technical problems may cause anxiety or helplessness in patients and caregivers. Strategies to mitigate this effect may include providing clear feedback when a task has been completed or a reading has been taken, avoiding time-pressured tasks, increasing task complexity gradually and within comfort, and using friendly, reassuring vocabulary. Moreover, systems could be designed to fulfill caregivers’ wishes to monitor the person with dementia’s location, physiological data and sleep while preventing the anxiety that could result from ‘abnormal normal’ and false readings, both of which can be common in elderly populations and in busy households.

Frustration and demotivation may also result from discrepancies between the expected function of an AT and its perceived usefulness (eg, caregivers not understanding why their smartwatch, adapted for the study, does not display the patient’s location). Fluctuating cognition can be a significant barrier to the remote monitoring of isolated patients as forgetting about one’s motivations to be monitored can lead to anxiety and agitation for being watched and, consequently, the disablement or destruction of equipment.

#### Aligning Tasks to the Patient’s Routine

Allowing patients to perform tasks or take readings at their own pace and in their preferred time prevents feelings of being forced into a routine. Completing short, finite tasks motivates people with dementia more than partial progress toward a complex goal, and short but frequent engagement forms both habit and skill. Patients with advanced dementia, however, may not have the patience, ability, or motivation to draw satisfaction from completing tasks. They may be more compliant to sporadic, in-depth, episodic checks than to daily routines that demand their sustained engagement. Moreover, it is advisable to introduce ATs that are in line with the patient’s existing habits and entertainment activities for higher engagement.

## Discussion

### Principal Findings

We explored functional and psychological needs of people with dementia using participatory user-centered design methods that produced a rich understanding of their experiences. These were expressed as design personas that help develop the empathy required for design and identify the challenges and opportunities of assistive remote monitoring technologies. Specific opportunities can subsequently be translated into technological innovation, public health strategy, and clinical practice through more focused user-centered design activities.

### Supporting the Translation of Stakeholder’s Experiences Into Public Health Strategy and Clinical Practice

Public health research and innovation processes benefit from involving patients and the public [41,57]. Our triangulation of findings with clinical, research, and organizational stakeholders enabled the definition and prioritization of care objectives, challenges, and both wide-ranging and solution-specific opportunities.

Moreover, the triangulation of findings with numerous stakeholders can significantly deepen researchers’ knowledge of relevant themes and reveal new opportunities, especially when stakeholders (eg, clinicians) are specialized in understanding patients’ needs. There are commonalities between the needs identified by patients and caregivers and the priorities identified by professional stakeholders (OT, health care service directors, clinical psychologists, and researchers in neuropsychiatry, behavior, and engineering). Thoroughly investigating these perspectives through 9 substudies elicited a wide variety of themes. The needs described by patients and caregivers mostly referred to physical health and independence in ADLs and started to reveal underlying values including autonomy, dignity, competence, relatedness, and reassurance. By contrast, clinicians identified more technology-specific (eg, “filtering ‘abnormal normal’ readings before alerting caregivers”) or medical (eg, “validated cognitive assessment tools”) needs and value-aligned ways to address the challenges that prevent the satisfaction of people with dementia and caregivers’ ADL needs. For example, “educating older adults to use the proposed technology” or “diagnosing and treating paranoia *before* prescribing a smart home system.”

The *personas* and the *needs map* helped highlight the range of needs within the dementia population. Personas emphasize that the ways users receive care and interact with smart home systems depend heavily on their socioeconomic status, health factors, care needs, technology usage, daily life routine, family dynamics, and support network within the community. All these factors impact how engaged they are with the technology, and therefore how much users value the system. It is hence important for participatory activities to investigate not only the prima facie content of personas, but also what elements or traits should be included within personas. The patient–caregiver personas also highlight the technical challenge of designing for a range of different home environments, for example, determining how many sensing devices are needed in each home.

Harnessing personas as case studies successfully elicited a wide range of responses from the clinicians, service managers, and researchers participating in this study’s final workshop. In our focus groups, some NHS service providers suggested refining these personas into a clinically accurate, quantitative, and validated spectrum of traits, contexts, health conditions, and stages of disease progression as an opportunity for further studies. This may be of value for researchers, designers, and engineers in a field where variables such as technology literacy, language, ethical and cultural differences, education, and the types of cognitive impairment (which may be related to memory, language, special acuity, sensory-motor, executive functioning, etc.) have an active impact not only on patient’s technology acceptance but also on the results of the cognitive and functional assessments upon which care plans are based. These variables can be investigated in further participatory activities involving people with dementia, caregivers, and clinicians. More precise clinical information can be identified by health care providers and through a review of the literature. However, the generalization of personas into a detailed characterization of social groups has been criticized [58]. While communicating fictional user archetypes can support empathy in design workshops, personas’ inherent risks of stereotyping, stigmatization, and limited diversity make them unsuitable as accurate representations of a population.

Our strategy of involving both participants who are early adopters of remote monitoring technologies and stakeholders more representative of the general population helped investigate both technology-specific considerations and more general needs and objectives in ADLs. We recommend that future studies replicate this strategy of combining evaluations of the technology-related experiences of early adopters (selected patients) with bottom–up investigations of the ADLs and care needs of the general population (members of the public and arbitrarily selected patients).

Conducting substudies in 3 phases and structuring Phases 02 and 03 around themes and personas identified in previous substudies allowed researchers to generalize insights elicited by investigating specific interactions with technology into widely relevant ADL needs and psychological factors. Conversely, conducting generative research and ideation activities based on previously defined patient and caregiver needs enabled researchers to guide stakeholders and people with dementia and caregivers to explore a wider design space and converge into more comprehensive and relevant service design and technology development opportunities.

Future studies continuing to combine results from a variety of stakeholders should ensure to evaluate findings across relevant groups of stakeholders to account for the potential limitations of one group speaking for another group, which may in fact disagree (eg, [44]). Therefore, to build on our findings, future studies can evaluate and explore each technology and service development opportunity identified by clinicians, researchers, and managers through the perspectives of a range of people with dementia and caregivers. Moreover, future studies may investigate how the prioritization of needs of people with dementia outlined in Table 1 and in the personas is dependent on the stage of dementia and on who is describing the problems.

### Methodological Limitations

Methodological limitations of this study should be addressed in future activities of research and development of “Healthy Homes.” While findings of ethnographic observations informed all the substudies, their documentation was incomplete due to operational constraints. Moreover, interviewing patients before and after they experience smart home interventions may reveal different insights than the sample of early adopters interviewed in this study. Comparisons would result in a more comprehensive understanding of how users’ preconceived ideas affect adoption and engagement with the technology. Quantifying the occurrence of each persona’s traits, conditions, and environments will require further studies. Although sample numbers were small, they are considered sufficient for qualitative analyses.

Our sampling strategy for the substudies of Phases 01 and 02 was to include both (1) end users and stakeholders who are early adopters of the CR&T’s smart home systems; and (2) patients, caregivers, clinicians, researchers, and managers that are representative of the wider public health “users,” services, and organizational processes. Phase 03’s substudy of the implementation of a more intensive monitoring system, however, could only be conducted under the current UK DRI CR&T’s research ethics approval and within suited recruitment timeframes with a cohort of self-selected UK DRI smart home trial users. Although this cohort was representative of the general dementia population when recruited through communities and social care channels for the UK DRI trial, participants may now be familiar with smart home technologies devices and inclined to support research. The samples might not be reflective of the common situations of disengaged, isolated people with dementia we identified in Phase 01 substudies and communicated in the “Patient–Caregiver Personas” section. Future qualitative studies investigating such intensive cognitive testing, smart home systems, and wearable-based monitoring may benefit from allocating sufficient time and resources to receive ethical approval to recruit a sample that comprises the needs of our personas. This would enable the analysis of their needs both before and after the implementation of smart home systems. Capturing the values and emotional expectations of people with dementia and caregivers who are living independently but anticipate they may need such monitoring systems in the future can aid researchers to address these factors through design. This could prevent the perceived utility of such systems from decreasing with actual use [14].

Despite this potential sampling bias, the cognitive testing tablets and the activity tracking smartwatches we introduced for Phase 03 were very unfamiliar for 8 of the 10 households. This unsurprisingly resulted in generally low acceptance and compliance, as may be expected in the general dementia population. Moreover, as the sample (10 caregivers and 2 people with dementia) was too small for quantitative analysis, the richness of insight resulting from the interviews’ thematic analysis was satisfactory for the purpose of our substudy. Interviews with this sample, however, had to be conducted via telephone due to the pandemic. This excluded 8 people with moderate and advanced dementia to be able to directly express their experiences. When in-person participatory design session return to be a possibility, creative and interactive activities during home visits can be more inclusive to people with dementia. The presence, sounds, aesthetics, and materials of prototypes can be used as props for creating and sharing concepts (eg, [54]).

### Limitations of Findings

Our strategy of recruiting early adopters of smart home systems for our substudies evaluating specific interactions with such technologies may have resulted in the underrepresentation of disengaged, nontechnology-savvy people with dementia and caregivers. Disengaged attitudes toward technology-based care are common in the general population of people with dementia and elderly caregivers, as identified in Phase 01 substudies and communicated in our personas. The challenges that the constant surveillance of smart home technologies poses around privacy [12] and agency [9] were not emphasized in our evaluative substudies as much expected [11]. Such themes were only touched on superficially by 2 nontech-savvy participants of Phase 03 interviews and by clinicians in our persona-based *Pan London OT Network* workshop.

Recruiting participants that represent the variety of attitudes toward care and technology outlined in our personas (including, for example, weariness toward devices, reluctance to obey automated alerts, reluctance for anyone to “know my business,” and isolated living situations) should be a priority of future sampling strategies. Best practices in conducting research with socially isolated older adults [46] should be followed. Understanding the human values (eg, dignity, autonomy) that underlie people’s attitudes toward smart homes can enable researchers to address tensions that may arise within a person or between stakeholders. For example, much of our cohort of early adopter caregivers inherently values “supporting research” and is inclined to data sharing, while our compliant people with dementia likely value “pleasing my caregiver.” The motivations of the general population should be understood in more detail for the translation of such products into public health pathways to be successful. In a context where products are often used *on* people, particular care must be taken in supporting end-users’ values to prevent undesirable but plausible consequences such as elderly abuse, loss of perceived autonomy or dignity, and increased isolation.

### Direct Implications of This Study’s Findings

Patient needs mapping results and personas are being used as a tool to communicate to the wider UK DRI research community the issues and challenges of creating environments that support independent living in an empathetic and realistic way. By improving such communication, this project aims to influence research and development on new AI and IoT technologies.

The challenges in delivering professional care and the technology development opportunities identified in this study are currently being addressed and prioritized by local and nation-wide public health care partners through the deployment of surveys. In parallel, our findings regarding intensive remote monitoring have directly informed the design of a new substudy by the CR&T and resulted in incremental improvements in the center’s cognitive testing app and its underlying clinical services. For example, the difficulty of puzzles now gradually increases, participants can pause tasks and repeat instructions, and feedback about tasks being completed is more explicit. Additionally, some of the insights that emerged from Phase 03 have been translated into improvements in the interface and user experience of the CR&T’s novel traumatic brain injury assessment app for in-person clinical use.

### Conclusions

Enabling communication between designers, technologists, and public health care providers (the UK DRI’s stakeholders) via participatory design processes and artifacts can foster more effective, inclusive, and rapid innovation in public health sectors. We aim to design and deploy remote monitoring and intervention systems that are fully integrated into a complex network of services, pathways, and stakeholders. Ensuring these systems are widely accessible yet tailored to the individual needs, technological knowledge, and level of engagement of individual patients and caregivers is a substantial task. Today’s pandemic-affected context has made it urgent to streamline innovation in this space through participatory, user-centered, and value-sensitive design.

Although this study focused on living with dementia, the iterative application of qualitative research methods involving patients, caregivers, and various stakeholders is applicable to other medical fields. This paper exemplifies how this methodology can reveal nuanced but critical psychosocial and contextual factors and support the development and translation of more patient-centered interventions.

## Abbreviations

ADLs: activities of daily living
AI: artificial intelligence
AT: assistive technologies
CR&T: Care Research and Technology Centre
DRI: Dementia Research Institute
IoT: internet of things
NHS: National Health Service
OT: occupational therapist
SABP: Surrey and Borders Partnership
